# Epidemiological trends and climate-driven dynamics of children respiratory infections in Lanzhou, China: a multi-pathogen time-series analysis

**DOI:** 10.3389/fcimb.2026.1777468

**Published:** 2026-05-19

**Authors:** Biao Wang, Hui Zhang, Shu Liang, Huan Wei, Lianmei Zhang, Miao Wang, Huimin Zhang, Xiaoshu Zhang, Maoxing Dong

**Affiliations:** 1 Gansu Provincial Center for Disease Control and Prevention, Lanzhou, China; 2 Public Health School, Gansu University of Chinese Medicine, Lanzhou, China; 3 Microbiology Laboratory, Lanzhou Center for Disease Control and Prevention, Lanzhou, China

**Keywords:** children, climate sensitivity monitoring, distribution lag nonlinear model, epidemiology, meteorological factors, respiratory infections

## Abstract

**Objective:**

The COVID-19 pandemic significantly affected the transmission of respiratory pathogens worldwide. This study aimed to describe the epidemiological characteristics of respiratory infections among children in Lanzhou, China, during the post-pandemic period and to investigate how meteorological factors influence the transmission of respiratory pathogens in the Lanzhou region.

**Methods:**

During the study period, 42.5% of children tested positive for respiratory pathogens (32.8% single, 9.7% mixed infections). Predominant pathogens were influenza virus (IFV, 12.3%), adenovirus (HAdV, 8.2%), rhinovirus (RV, 7.5%), and respiratory syncytial virus (RSV, 6.7%). RSV and IFV peaked in infants <1 year (14.1% and 13.5%), while Mycoplasma pneumoniae (MP) was highest in school-aged children (8.5%). Mixed infections increased with age (2.8% to 11.6%), with no sex differences. Seasonal patterns varied: IFV peaked in winter (26.9%), enteroviruses in summer (18.9%), and RSV showed bimodal winter-spring distribution. IFV negatively correlated with temperature (r = -0.50) and positively with atmospheric pressure (r = 0.37). RSV negatively correlated with temperature (r = -0.49), while MP positively correlated with humidity and pressure.Nonlinear lag modeling showed immediate meteorological effects (0-week lag). IFV showed elevated risk under extreme low temperatures and high pressure (RR: 10.395 and 18.597), though wide confidence intervals warrant caution. HAdV and MP risks increased similarly. Conversely, HPIV and RV showed protective associations at low temperatures or high wind speeds. Wind speed appeared protective against most pathogens, but this observational finding requires interventional validation.

**Conclusion:**

Pediatric respiratory pathogen prevalence in Lanzhou exhibited distinct age-dependent and seasonal characteristics, with pathogen-specific meteorological associations. Detection risks for IFV, RSV, HAdV, and MP increased with low temperatures and high pressure, while RV and HPIV showed opposite patterns. The protective wind speed effect suggests ventilation improvements may reduce transmission risk, though this hypothesis-generating conclusion requires further validation. These findings inform age-stratified, seasonally-adapted prevention strategies and meteorological early warning systems.

## 1 Introduction

Acute respiratory infections (ARIs) are a leading cause of morbidity and mortality in children and infants worldwide. It is estimated that approximately 80% of ARIs are viral (Choi et al., 2018). In China, ARIs continue to account for a substantial number of pediatric outpatient visits and hospitalizations Cui et al., 2024), imposing heavy burdens on the public health system. RSV is the leading pathogen causing lower respiratory tract infections in infants and young children (25.7%), followed by RV (17.4%), HPIV (15.8%), IFV (14.2%), and HAdV (10.7%) (Li et al., 2021). The common bacterial pathogen, MP, is more prevalent among school-aged children (China Medicine Education Association Committeeon Pediatrics, 2023). Studies across China have indicated significant regional variations in pathogen prevalence patterns (Huang et al., 2025; Wang et al., 2025; Jie et al., 2025), making it crucial to understand local epidemiological characteristics to develop targeted public health strategies. Environmental factors, particularly meteorological conditions, significantly influence respiratory pathogen circulation. Temperature, humidity, and other meteorological factors play key roles in viral environmental survival, transmission efficiency, and host susceptibility (Lapeña et al., 2005; Tang and Loh, 2014; Sundell et al., 2016; du Prel et al., 2009; Pan et al., 2017; Chen et al., 2014; Darniot et al., 2018). Previous studies have indicated that specific meteorological conditions, such as low temperatures and low relative humidity, may promote or suppress the transmission of respiratory pathogens, including IFV, RSV, HAdV, and HPIV (Xu et al., 2021). However, such associations often exhibit nonlinearity and temporal lag, necessitating the application of time-series analysis methods to comprehensively capture their complex effects.

Lanzhou City is located in central Gansu Province, northwest China (36°03′N, 103°40′E), making it an ideal region for studying the relationship between climate and respiratory pathogen transmission. The city is situated within a narrow river valley basin surrounded by mountains, exhibiting a depth-to-width ratio of approximately 0.07 and typical features of an enclosed mountain basin. Owing to topographical constraints, local wind speeds are low, with an annual calm wind rate as high as 62%. Isolated from maritime influences, the region lacks warm and humid air currents, resulting in sparse annual precipitation. Combined with abundant sunshine and high evaporation rates, this creates a typical temperate continental climate in the region. This unique combination of geographical environment and climatic characteristics makes Lanzhou an ideal research site for exploring the association between meteorological factors and the transmission of respiratory diseases. Since the global outbreak of coronavirus disease 2019 (COVID-19) in late 2019, countries have implemented NPIs, including social distancing, mask-wearing, and school closures, to curb the spread of severe acute respiratory syndrome coronavirus 2 (SARS-CoV-2) (Huang et al., 2021). While these measures altered environmental exposure patterns and reduced interpersonal contact, thereby suppressing the prevalence of multiple respiratory pathogens, they also resulted in the accumulation of immunity debt among children (Terliesner et al., 2022). Following the lifting of China’s dynamic zero-COVID policy in late 2022, many regions experienced explosive outbreaks of IFV, RSV, and MP. This suggests that the removal of NPIs may have reshaped the relationship between environmental factors and the risk of respiratory pathogen infection. Therefore, systematically reassessing the impact of meteorological factors on the transmission of pediatric respiratory pathogens in the post-pandemic era is of significant practical importance. Given this context, this study aimed to comprehensively describe the epidemiological characteristics of 11 pediatric respiratory pathogens SARS-CoV-2, IFV, RSV, HAdV, HMPV, HPIV, HCoV, HBoV, RV, EV, and MP) and elucidate the influence of meteorological factors on their transmission. This study employed a combined approach of GAMs and DLNMs to systematically evaluate the nonlinear and lagged effects of meteorological factors—including temperature, humidity, atmospheric pressure, and wind speed—on pathogen detection rates. The findings will provide scientific evidence for establishing an early warning system for respiratory infectious diseases based on meteorological conditions and guide the development of targeted public health interventions to reduce the burden of these diseases.

## 2 Materials and methods

### 2.1 Respiratory multipathogen surveillance data

The data for this study were derived from respiratory multi-pathogen surveillance data collected in Lanzhou City from January 2023 to June 2024 as part of China’s 13-pathogen respiratory pathogen real-time surveillance program and from respiratory multi-pathogen surveillance data in Lanzhou City from July 2024 to May 2025, as recorded in China’s Influenza Surveillance Information System. The inclusion criteria for cases in the surveillance data were as follows: ① acute infection symptoms (meeting any of the following criteria): fever, chills, and abnormal white blood cell count (decreased or increased); adults (≥16 years): (4.0–10.0) × 10^9/L; children (6–15 years): (4.5–13.5) × 10^9^/L; children (1–5 years): (6.0–15.5) × 10^9^/L; ② clinical symptoms (meeting any of the following criteria): runny nose, cough with sputum, wheezing, swelling or pain in the throat or larynx, chest tightness or pain, fatigue, abdominal pain, or diarrhea. Qualified medical personnel at sentinel hospitals must strictly follow the monitoring protocol to collect nasopharyngeal swab samples and simultaneously gather the corresponding epidemiological data. Samples were sent immediately to the Lanzhou Center for Disease Control and Prevention for respiratory pathogen nucleic acid testing. Samples submitted within 24 h should be stored at 4 °C, and samples submitted after 24 h should be stored at -70 °C.This study included data from 1,502 nasopharyngeal swab tests conducted on pediatric patients. This dataset includes weekly testing volumes and detection rates of respiratory pathogens in Lanzhou. The positivity rate for each respiratory pathogen was calculated using the following formula:


$$\text{Positive Rate}(\%)=\frac{\text{Npositive}}{/\text{Ntested}}\times 100\%$$

### 2.2 Meteorological data

Meteorological data are publicly accessible after registration on the official website of the National Meteorological Science Data Center (https://data.cma.cn/). The meteorological factors included the weekly average temperature (°C), weekly average temperature difference (°C), weekly average air pressure (hPa), weekly average precipitation (mm), weekly average relative humidity (%), weekly average sunshine duration (h), and weekly average wind speed (m/s).

### 2.3 Data preprocessing

Respiratory multi-pathogen surveillance and meteorological data were integrated into a weekly time series for subsequent analysis. Weekly meteorological data were matched with the positivity rates of each pathogen in the study. Prior to modeling, all time series underwent data missingness checks to ensure consistency across datasets.

### 2.4 Data analysis

Data processing and statistical analyses. Descriptive statistics were first used to calculate the overall positivity rates for 11 respiratory pathogens, stratified by age group (<1, 1–3, 3–6, 6–11, and >11 years) and sex. Categorical variables were expressed as frequencies and percentages. Comparisons between categorical variable groups were performed using chi-square or Fisher’s exact tests. *Post-hoc* multiple comparisons were corrected using the Bonferroni correction. The correlation between pathogen positivity rates and meteorological factors was assessed using Spearman’s rank correlation analysis. To avoid multicollinearity issues, a comprehensive multicollinearity diagnosis was performed for all meteorological variables, including the calculation of the variance inflation factor (VIF) and assessment of the condition index. The correlation results were visualized using histograms, locally estimated scatter smoothing (LOESS)-smoothed scatter plots, and correlation matrix plots. The significance level was set at P < 0.05. Distributed lag nonlinear models (DLNM) were employed to assess the lagged effects of meteorological factors on respiratory pathogen detection rates. The DLNM simultaneously captures nonlinear exposure-response relationships and the temporal distribution of lagged effects. The model specification is as follows:


log (E(Yt)) = α + cb (Meteorological_t, l, df, lag=0-4) + ns(Time_t, df) + factor (Season_t) + log(Total_samples_t).

where Yt denotes the number of pathogen detection cases in week t, cb() represents cross-knot functions modeling the exposure-lag-response relationship of meteorological variables, ns() denotes natural spline functions controlling long-term trends, and Season_t controls seasonal effects. Total_samples_t is the log-shift of the total detection count. The model employs a quasi-Poisson regression framework to correct for excessive dispersion in weekly case counts. Natural cubic spline functions were used as the basis for the functions. For the exposure dimension, nodes were set at the 10th, 75th, and 90th percentiles of air temperature and relative humidity (df = 3) and at the 25th and 75th percentiles of the remaining meteorological variables (df = 2). Boundary nodes were set at the minimum and maximum observed values of each variable, and lag-dimension nodes were placed at equal intervals on a logarithmic scale within the maximum lag of four weeks (df = 3 for temperature/humidity; df = 2 for other variables). We extracted estimates for the following three types of effects: immediate effect (lag 0), representing the immediate impact of exposure in the current week; peak effect, representing the maximum effect across the entire lag period and its corresponding lag time; and cumulative effect, representing the overall effect across the entire lag period (lags 0–4). All effects are presented as relative risks (RR) with 95% confidence intervals, using the median of each exposure variable as a common reference point. Significance was defined as a 95% confidence interval that did not contain 1.0. The relatively high RR values observed under extreme exposure conditions are due to the fact that these outliers are located at the tail end of the observed data distribution. Data sparsity leads to reduced estimation stability of the spline function at the boundaries, resulting in potentially inflated point estimates accompanied by wider confidence intervals. All analyses were conducted using the dlnm and mgcv packages in R software (vresion4.5.3).

## 3 Results

### 3.1 Epidemiological characteristics of respiratory pathogens in children in Lanzhou city, China

**Figure 1:**
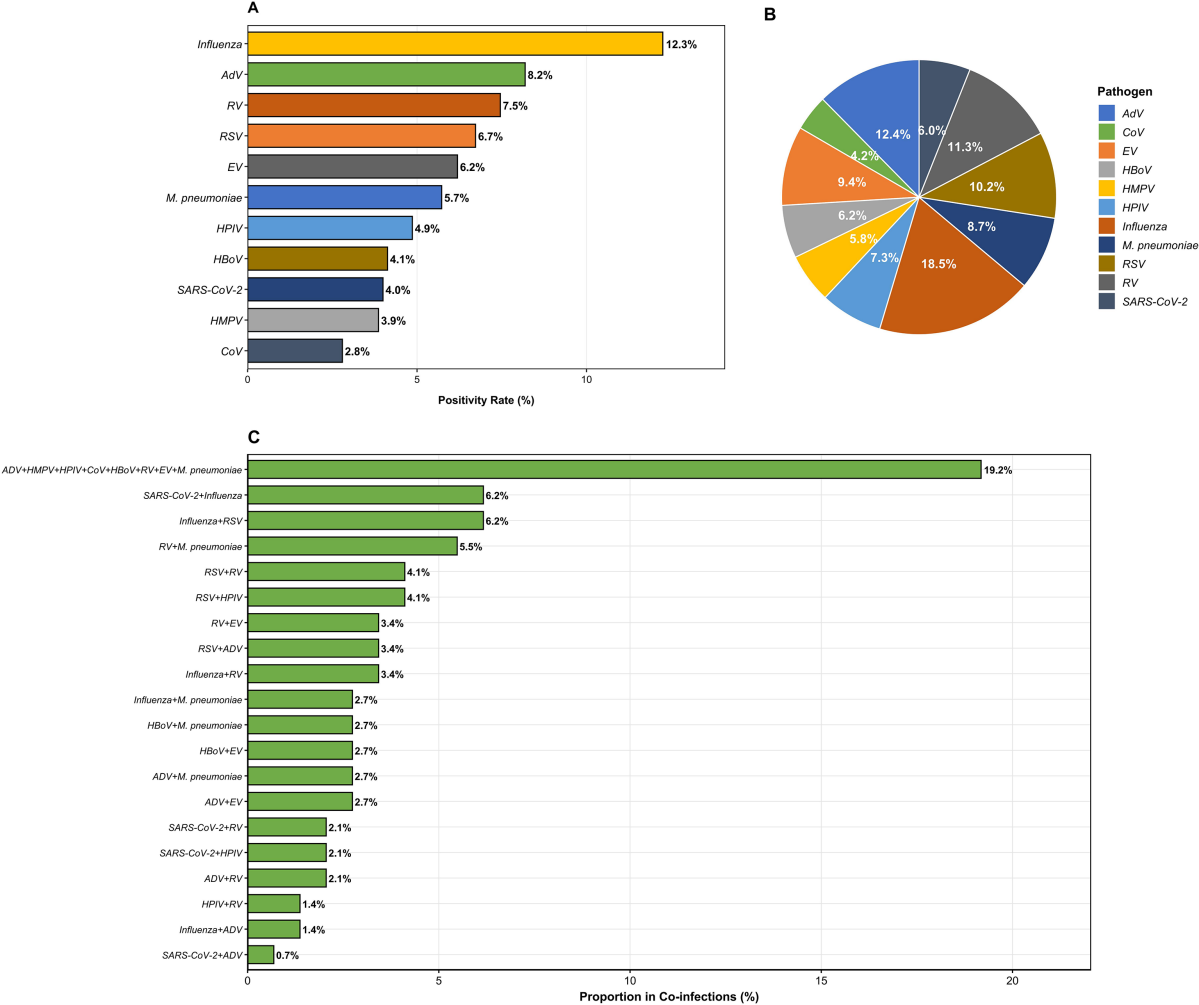
**(A)** Positivity rates for respiratory pathogens in mono-infections. **(B)** Distribution of pathogens in mono-infections. **(C)** Leading co-infection combinations.

During the study period, the overall positive rate for respiratory pathogens among children in Lanzhou was 42.5%, with single-pathogen infections accounting for 32.8% and mixed infections accounting for 9.7%. Among single-pathogen infections, the top four pathogens detected were IFV, 12.3%, HAdV, 8.2%, RV, 7.5%, and RSV, 6.7%, whereas HCoV had the lowest detection rate (2.8%) (**Figure 1A**). Regarding pathogen composition (**Figure 1B**), RSV accounted for the largest proportion (18.5%) of all positive detections, highlighting its significant role in pediatric respiratory infections, whereas SARS-CoV-2 had the smallest proportion (6.0%), reflecting its relatively limited prevalence during the study period. Analysis of mixed infection patterns (**Figure 1C**) revealed that complex co-infections involving multiple pathogens (HAdV, HMPV, HPIV, HCoV, HBoV, RV, EV, and MP) were the most common, accounting for 19.2% of all mixed infections. Among dual-pathogen co-infections, the SARS-CoV-2 + IFV combination was the most prevalent (6.2%), followed by IFV + RSV (5.5%) and RV + MP (4.1%), suggesting potential overlap or synergistic effects among pathogens in terms of transmission seasonality and host susceptibility.

### 3.2 Age and gender distribution characteristics of detection rates for respiratory pathogens

**Figure 2:**
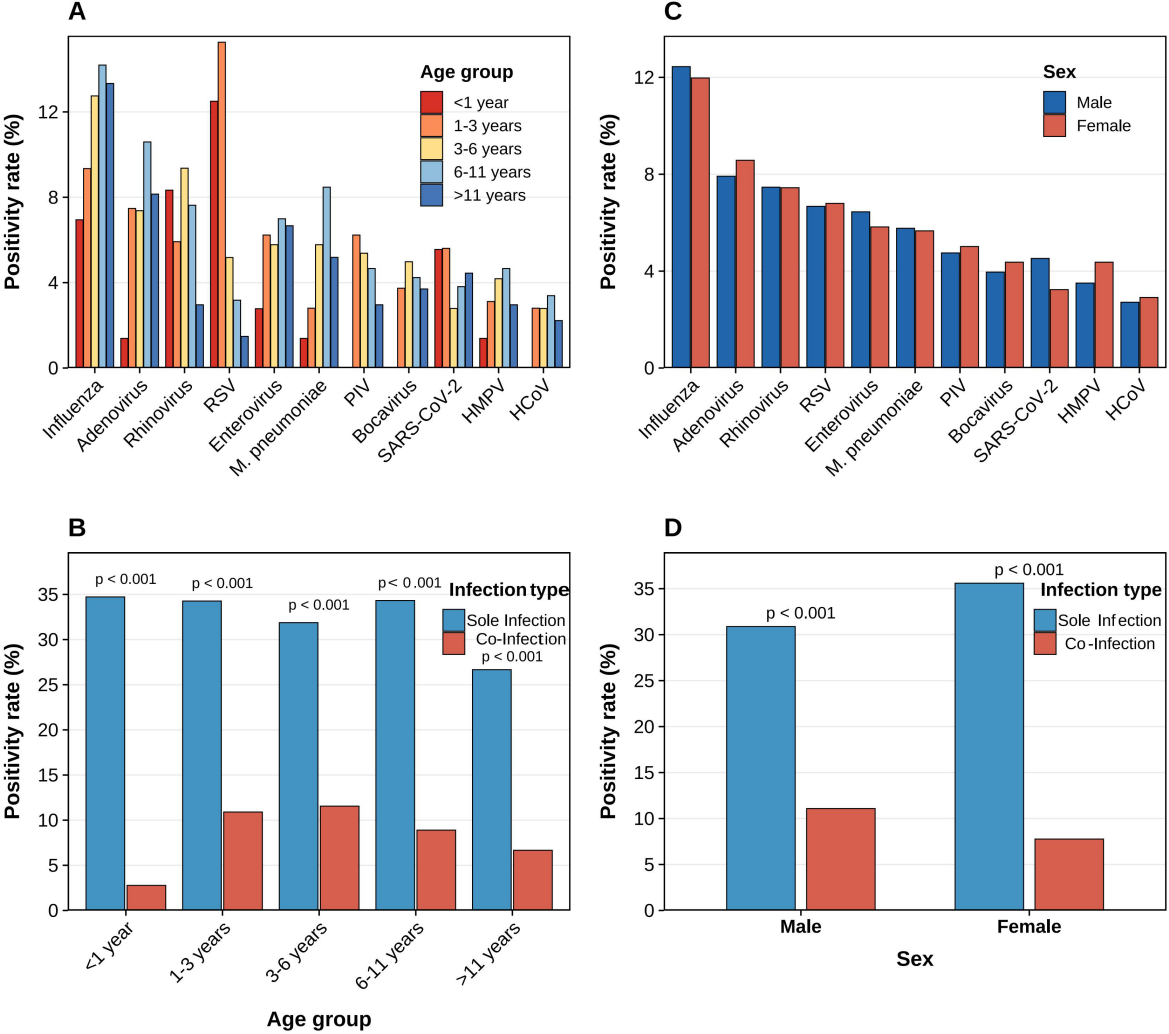
Age and sex stratification of respiratory pathogen detection rates **(A)** Pathogen positivity rates by age group; **(B)** Infection type distribution by age; **(C)** Pathogen positivity rates by sex; **(D)** Infection type distribution by sex. Sole infections were significantly higher than co-infections in all groups (P < 0.001).

Age-stratified analysis revealed significant differences in the prevalence of respiratory pathogens across the age groups (**Figure 2A**). IFV exhibited the highest positivity rates in infants (<1 year, 13.5%) and adolescents (>11 years, 13.2%), demonstrating a bimodal distribution. RSV infection was predominantly concentrated in infancy and early childhood, peaking in the <1-year-old group (14.1%), with a significant decline in the detection rates with increasing age. MP exhibited the highest detection rate among school-aged children (6–11 years, 8.5%), consistent with its epidemiological characteristics of high transmission efficiency in group settings. RV maintained high detection rates across all age groups, most notably in the 1–3-year-old cohort (10.2%), indicating its persistent transmission capacity as a common pathogen in the community. In contrast, HCoV, HBoV, and HMPV exhibited low positivity rates (<5%) across all age groups. Analysis of the infection types revealed that single infections significantly outnumbered mixed infections across all age groups (P<0.001) (**Figure 2B**). The <1-year-old group exhibited the highest single infection rate (35.1%) and the lowest mixed infection rate (2.8%) among all groups. With increasing age, the single infection rate declined to 26.8% in the >11-year-old group, whereas the mixed infection rate increased progressively. The mixed infection rates in the 1–3, 3–6, and 6–11 age groups were 10.9%, 11.6%, and 9.1%, respectively, all significantly higher than those in the <1 year group (P<0.001). This suggests that expanding social contact networks and increased exposure heterogeneity may be key drivers of the rising co-infection risk among preschool and school-aged children. Gender-stratified analysis revealed no significant differences in positivity rates for most pathogens between the sexes. Influenza virus detection rates were similar in males (12.8%) and females (12.1%); adenovirus rates were slightly higher in males (8.9% vs. 8.2% in females); and RSV distribution was comparable between the sexes (7.4% vs. 7.3%). Other pathogens, such as MP, HPIV, and HBoV, showed no statistically significant sex differences (**Figure 2C**). Gender comparisons of infection types (**Figure 2D**) revealed that both single-pathogen and mixed infection rates were significantly higher in males than in females (both P<0.001). The single infection rate among females (35.6%) was slightly higher than that among males (31.2%), while the mixed infection rate was similar between the sexes (11.2% for males, 7.8% for females), with no statistically significant difference. In summary, the prevalence of respiratory pathogens among children in Lanzhou exhibited distinct age-dependent patterns: infants and toddlers predominantly experienced single infections and were more susceptible to RSV and IFV, whereas preschool- and school-aged children showed a significant increase in mixed infections. Sex had no significant impact on pathogen detection rates or infection patterns.

### 3.3 Seasonal distribution of respiratory pathogens

**Figure 3:**
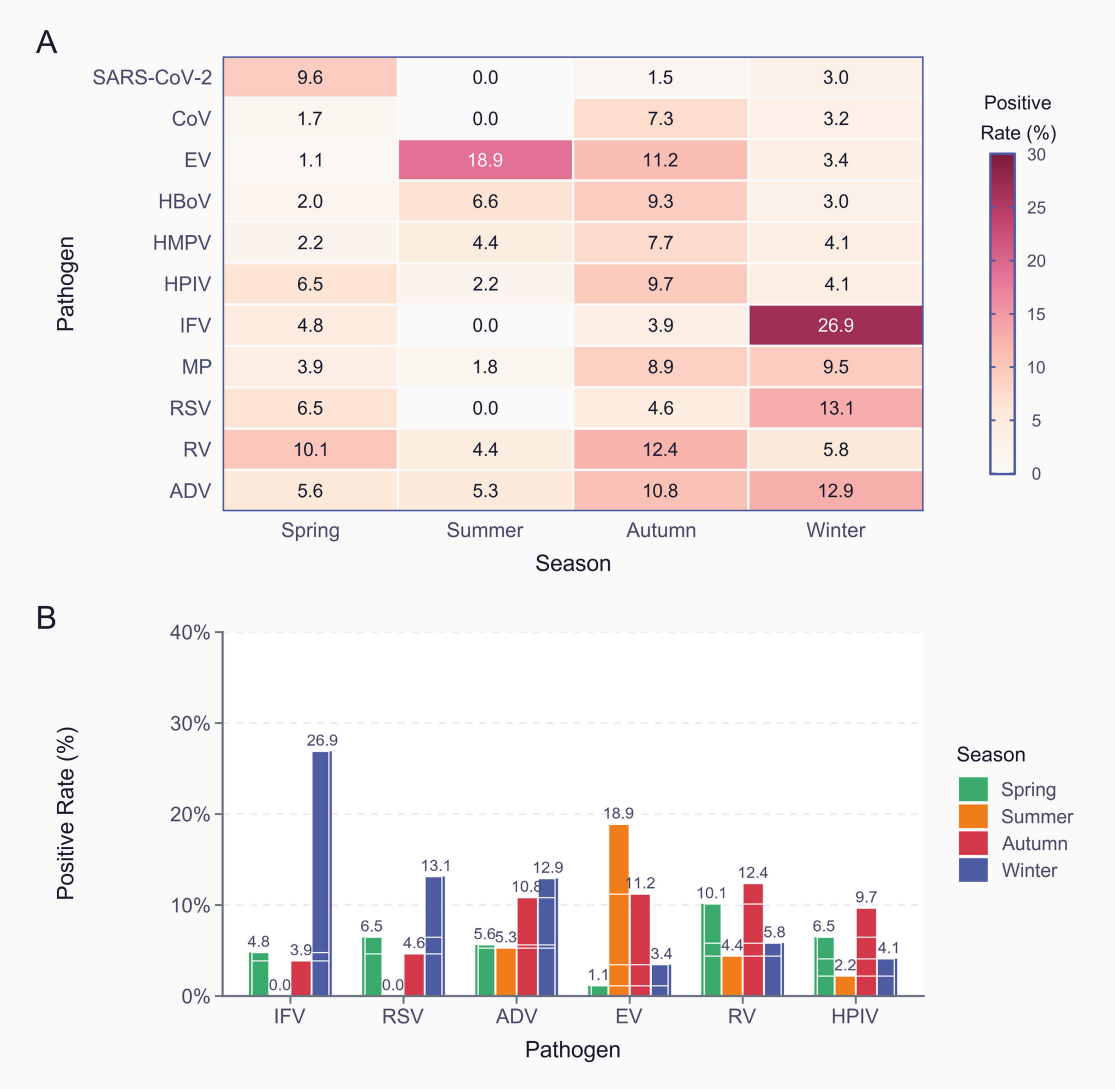
Seasonal patterns of respiratory pathogen positive rates (May 2023 - April 2025). **(A)** Heatmap of 11 pathogens across seasons. **(B)** Seasonal comparison of major respiratory pathogens.

The seasonal distribution patterns of respiratory pathogens exhibited marked variability across different seasons (**Figures 3A, B**). Among the 11 respiratory pathogens analyzed, IFV showed the most pronounced seasonal variation, with the highest positivity rate in winter (26.9%), followed by spring (4.8%), while activity was minimal in summer and fall (undetected and 3.9%, respectively). EV exhibited the opposite seasonal pattern, peaking in summer (18.9%) and followed by autumn (11.2%), whereas detection rates were significantly lower in spring (1.1%) and winter (3.4%). This pattern aligns with the typical summer and autumn epidemics characteristic of enteroviruses. Respiratory syncytial virus (RSV) exhibited a bimodal distribution, peaking in winter (13.1%) and spring (6.5%) and not detected in summer. RV maintained relatively stable circulation throughout the year, with the highest detection rate in autumn (12.4%) and the lowest in summer (4.4%). HAdV exhibited continuous year-round circulation, with relatively uniform positivity rates across seasons, ranging from 5.3% in summer to 12.9% in winter. HPIV activity peaked in the fall (7.7%) and had reduced circulation in summer (2.2%) and winter (4.1%). Among pathogens with lower prevalence, HBoV peaked in the fall (9.3%), whereas HMPV had the highest detection rates in the fall (7.7%) and summer (4.4%). MP exhibited peak activity in fall (8.9%) and winter (9.5%). The heat map (**Figure 3A**) clearly illustrates the seasonal clustering patterns of different pathogens: winter is characterized by high activity of IFV and RSV, summer is dominated by EV, and fall serves as a transitional period with increased activity of multiple pathogens (including RV, HBoV, and HPIV). The data revealed distinct seasonal patterns among respiratory pathogens, including winter-dominant viruses (IFV and RSV), summer-dominant viruses (EV), and year-round circulating pathogens (HAdV and RV). These patterns represent different epidemiological characteristics and can provide a basis for developing targeted surveillance and prevention strategies for this disease.

### 3.4 Correlation analysis between respiratory pathogens and meteorological factors

**Figure 4:**
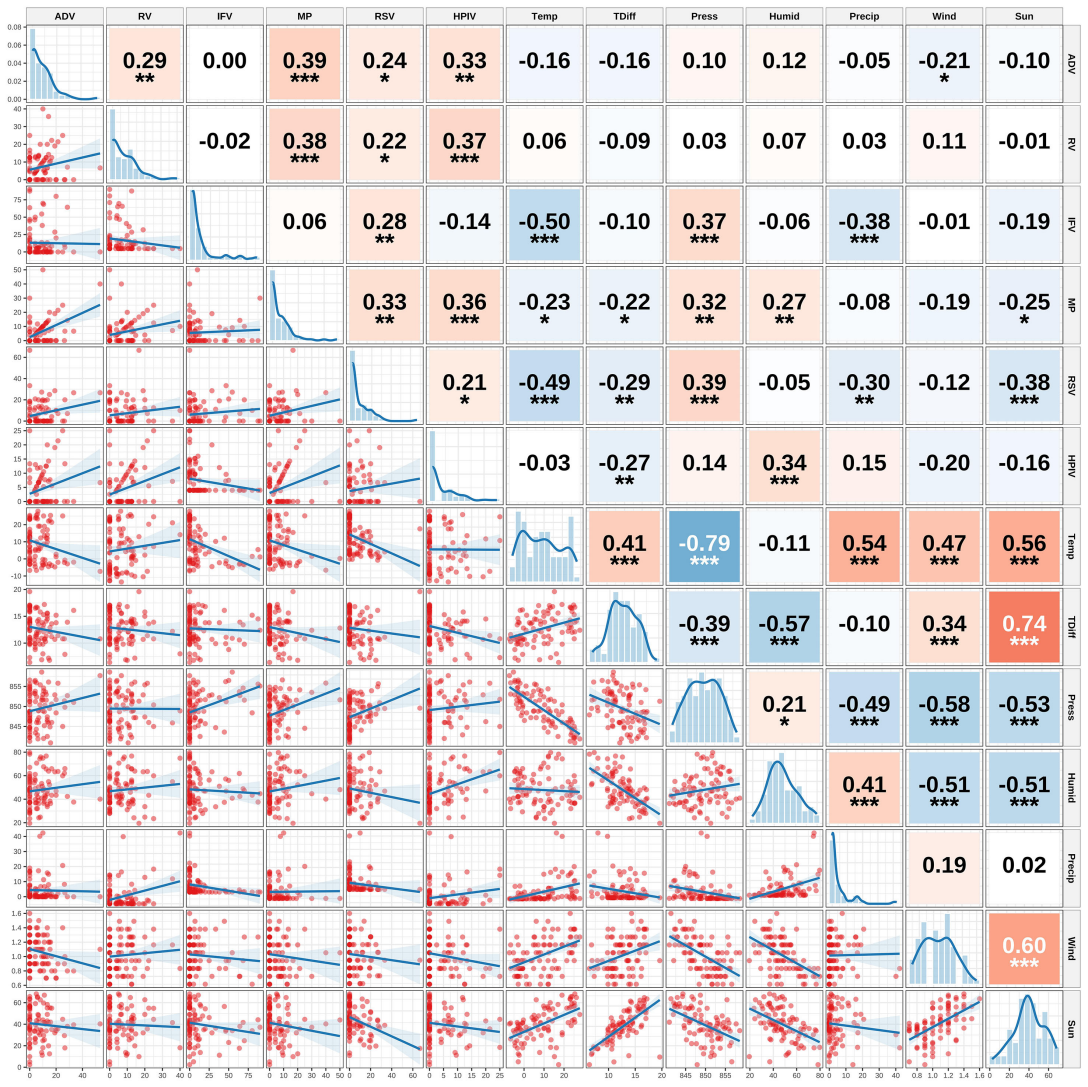
Correlation matrix between positivity rates of major respiratory pathogens and meteorological factors *indicate statistical significance levels: *p < 0.05, **p < 0.01, ***p < 0.001.

Through Spearman correlation analysis, we established relationships between the positivity rates of major respiratory pathogens (HAdV, RV, IFV, MP, RSV, and HPIV) and meteorological factors, including air temperature (Temp), daily temperature variation (TDiff), Atmospheric Pressure (Press), Relative Humidity (Humid), precipitation (Precip), Wind Speed (Wind), and Sunshine Duration (Sun) (**Figure 4**). The matrix displays histograms of the variable distributions along the diagonal. Scatter plots of paired variables with locally weighted regression (LOESS) fitted curves are shown below the diagonal. Above the diagonal are the Spearman correlation coefficients and their significance levels.

Internal Correlation Structure of Meteorological Factors: Analysis revealed a distinct internal correlation structure among meteorological factors. Air temperature was significantly negatively correlated with air pressure (r = −0.68, P < 0.001) and positively correlated with relative humidity (r = 0.45, P < 0.001). Relative humidity was positively correlated with precipitation (r = 0.52, P < 0.001). This suggests that changes in a single meteorological indicator are often accompanied by coordinated fluctuations in other meteorological elements, reflecting the holistic characteristics of the system. However, the multicollinearity assessment revealed that the VIF values ranged from 1.71 to 3.88, well below the generally accepted threshold of 5, indicating a low level of multicollinearity. See in **Supplementary S1**.

Correlations among pathogens: Among the respiratory pathogens, some exhibited varying degrees of positive correlation. The most significant correlation was observed between HAdV and RV (r = 0.42, P < 0.01), suggesting that they may share similar seasonal drivers or exhibit synergistic transmission patterns during specific periods.

Association patterns between pathogens and meteorological factors: The prevalence of pathogens showed pathogen-specific significant correlations with the meteorological factors. IFV was significantly negatively correlated with air temperature (r = −0.50, P < 0.001) and precipitation (r = −0.38, P < 0.01), and significantly positively correlated with atmospheric pressure (r = 0.37, P < 0.01), suggesting a preference for cold, dry, and high-pressure environmental conditions. RSV exhibited similar meteorological preferences, showing significant negative correlations with air temperature (r = −0.49, P < 0.001), daily temperature range (r = −0.29, P < 0.05), precipitation (r = −0.30, P < 0.05), and sunshine duration (r = −0.38, P < 0.01), and a positive correlation with atmospheric pressure (r = 0.39, P < 0.01). This further supports its epidemiological characteristics as a typical wintertime pathogen. MP exhibited distinct patterns of association with meteorological factors, showing positive correlations with relative humidity (r = 0.27, P < 0.05) and atmospheric pressure (r = 0.32, P < 0.05), suggesting enhanced transmission under mildly humid conditions with a relatively stable atmospheric pressure. HMPV showed a significant positive correlation with relative humidity (r=0.34, P<0.01) but a negative correlation with daily temperature range (r=−0.27, P<0.05), indicating that its transmission may rely more on humid conditions with minimal meteorological fluctuations. This correlation matrix visually depicts multidimensional association patterns among pathogens and between pathogens and meteorological factors, revealing differences in the “meteorological niches” occupied by various pathogens. This study provides a crucial theoretical foundation for further exploration of the mechanisms underlying seasonal infection patterns and potential synergistic or competitive relationships among pathogens.

### 3.5 Nonlinear association and lagged effects of meteorological factors on the detection risk of respiratory pathogens

**Figure 5:**
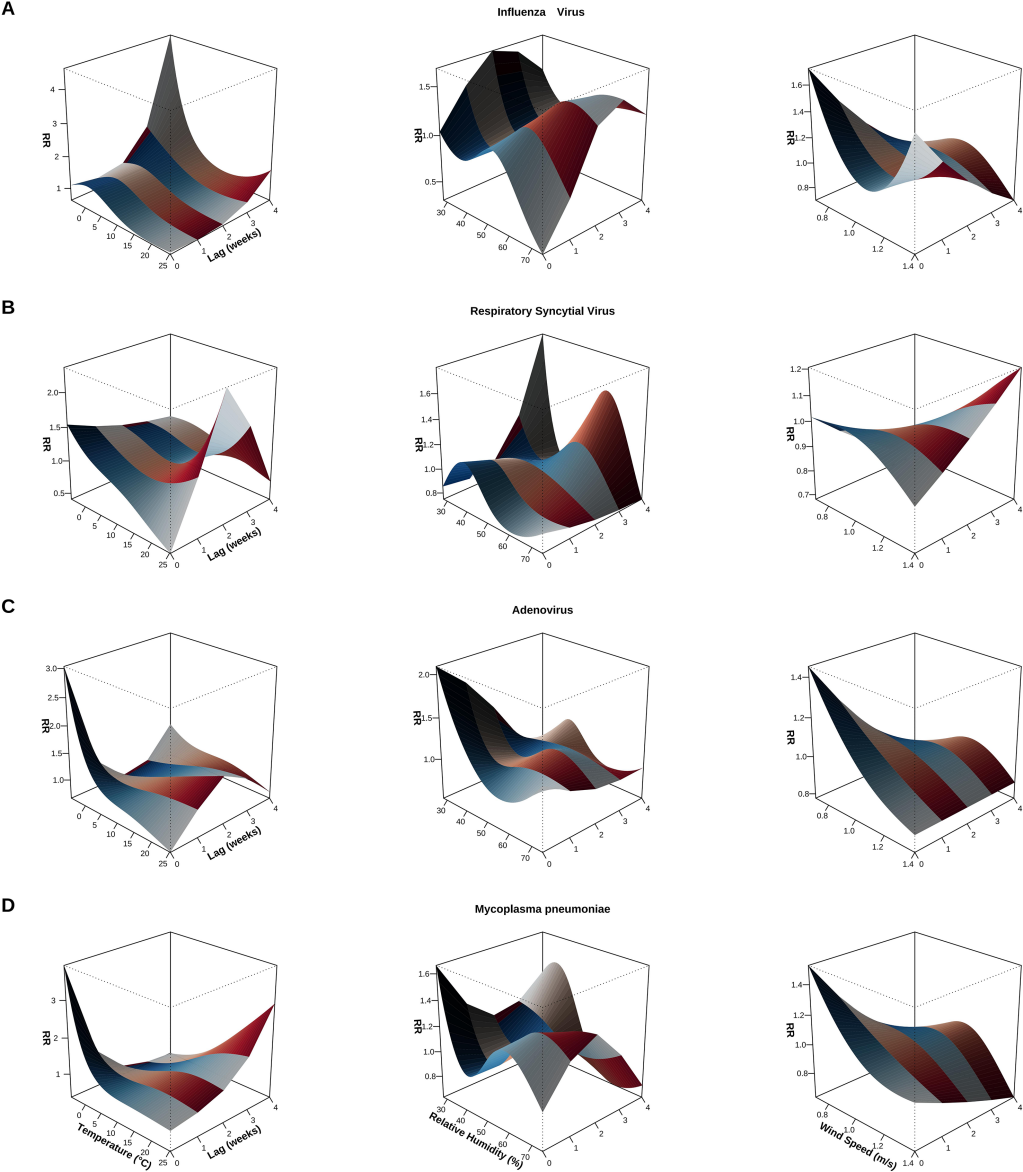
Three−dimensional exposure–lag–response surfaces showing the effects of meteorological factors on pathogen−specific detection risk. Rows **(A–D)** correspond to IFV, RSV, HAdV and MP, respectively. Columns (left to right) depict the combined effects of lag weeks and (1) temperature (°C), (2) relative humidity (%) and (3) wind speed (m/s) on the RR. Surface height and color indicate RR magnitude; peaks denote exposure–lag combinations associated with increased detection risk. Estimates derive from DLNM adjusted for long−term trend, seasonality and total sample volume (offset). Peaks shown represent immediate or short−term effects. Numerical peak estimates, 95% confidence intervals and sensitivity analyses are reported in the main text and **Supplementary Materials**.

Pathogen-specific exposure-response relationships: DLNM analysis revealed significant and pathogen-specific real-time associations between meteorological factors and detection rates of multiple respiratory pathogens (**Figure 5**; **Supplementary Figure 1**). Peak effects for all pathogens occurred at a lag of 0 weeks, indicating that meteorological factors primarily exert short-term effects within the same week on respiratory diseases. For IFV, the RR was 18.597 (95% CI: 3.591–96.320) under high barometric pressure (856.14 hPa) and 2.561 (95% CI: 1.147–5.715) at 56.3% relative humidity, and a high RR of 10.395 (95% CI: 1.650–65.480) at low temperature (−4.64 °C), indicating significantly elevated IFV infection risk under low-temperature and high-pressure conditions. HAdV similarly exhibited an increased risk at high barometric pressure (856.14 hPa), with an RR of 3.437 (95% CI: 1.492–7.920); at extremely low temperatures (−4.64 °C), the RR was 2.124 (95% CI: 1.098–4.107), suggesting sensitivity to cold environments. MP showed an RR of 12.472 (95% CI: 3.089–50.361) at high barometric pressure (856.14 hPa) and an RR of 3.847 (95% CI: 1.081–13.688) at moderate temperatures (17 °C). Notably, wind speed at higher levels (1.4 m/s) exhibited a significant protective effect with RR = 0.274 (95% CI: 0.085–0.885), suggesting that ventilation conditions may reduce MP transmission risk by diluting aerosol concentrations. HMPV exhibited an RR of 1.821 (95% CI: 1.068–3.105) at 56.3% relative humidity, indicating its preference for humid conditions. HPIV exhibited significant protective associations under low humidity (28.08%) and low temperature (−4.64 °C) conditions, with RRs of 0.169 (95% CI: 0.037–0.768) and 0.490 (95% CI: 0.247–0.969), respectively. The wind speed effect was also negative, with an RR of 0.162 (95% CI: 0.047–0.555) at 1.38 m/s. RV exhibited protective associations under high barometric pressure (855.34 hPa), and low temperature (−3.38 °C) conditions, with RRs of 0.229 (95% CI: 0.088–0.593) and 0.453 (95% CI: 0.253–0.810), respectively, and RR was 0.252 (95% CI: 0.117–0.544) at wind speeds of 0.8 m/s. In summary, viruses such as IFV, HAdV, and MP, as well as atypical pathogens, typically exhibit an RR > 1 under conditions of high atmospheric pressure or extreme temperatures, whereas pathogens such as HPIV and RV often exhibit an RR < 1 under conditions of low temperature, low humidity, or high wind speeds, reflecting the fundamental differences in the meteorological niches of various pathogens. It is worth emphasizing that wind speed exhibited a protective association (RR < 1) for most pathogens, suggesting that enhanced ventilation may be a common intervention strategy across pathogens for reducing the risk of respiratory infections. Three-dimensional exposure-lag-response surface analysis: **Figure 5** shows the three-dimensional exposure-lag-response surface linking meteorological factors to detection risks for four common pathogens (IFV, RSV, HAdV, and MP). Although peak effects for all pathogens occurred at lag 0 weeks (**Supplementary Figure 1**), the surfaces revealed distribution patterns of meteorological effects across the entire lag window (0–4 weeks) and pathogen-specific characteristics of the meteorological effects. The temperature-lag surface for IFV (**Figure 5A**) showed that RR peaks in the low-temperature range were concentrated at lag 0 weeks, with effects gradually diminishing during subsequent lag periods (i.e., 1–4 weeks). The humidity surface exhibited a peak at moderate humidity levels (50–60%) with weaker lag effects, and the wind speed surface remained generally flat, showing no significant lag pattern. This indicates that IFV’s response to temperature is primarily immediate, consistent with its role as a dominant winter pathogen. The RSV (**Figure 5B**) surface exhibited a pronounced risk peak at lag 0 weeks. However, secondary peaks remained observable at lag 1–2 weeks under conditions of moderately low humidity (30–50%) or specific temperature combinations (5–10 °C), suggesting that humidity and temperature may exert compound effects on RSV activity across different lag periods. However, the RR values for the secondary peaks were significantly lower than those at lag 0 (approximately 30–50% of the main peak) and exhibited wider confidence intervals, suggesting limited statistical significance. The response surface for HAdV (**Figure 5C**) showed a primary peak at lag 0 with a relatively flat overall surface, indicating milder meteorological influences on the HAdV. Mild RR variations were observed in subsequent lags under certain extreme wind speeds (>2.0 m/s) or humidity values (<20% or >80%), but none reached statistical significance, suggesting that HAdV exhibits relatively limited sensitivity to meteorological conditions. The surface for MP (**Figure 5D**) at lag 0 revealed significantly elevated infection risk (RR > 3) under certain temperature/humidity combinations (e.g., high pressure + moderate temperatures 15–20 °C), while exhibiting a protective effect (RR < 0.5) under other combinations (e.g., high wind speeds > 1.5 m/s or high humidity > 70%). This suggests MPs’ sensitivity to meteorological conditions is bidirectional and primarily manifests as an immediate response. During subsequent lag periods (1–4 weeks), the curve flattens without showing sustained or delayed effects. Overall, meteorological associations for all four pathogens were dominated by immediate effects (lag 0 weeks). Although secondary lag peaks are observable for some pathogens (e.g., RSV), their effect sizes and statistical significance are substantially lower than those of immediate effects. This temporal pattern aligns with the short incubation periods of these pathogens (typically 1–7 days) and the weekly aggregation of meteorological data, suggesting that early warning systems based on meteorological conditions should prioritize the immediate effects of meteorological exposure in the same week as the event.

## 4 Discussion

This study systematically evaluated the association between meteorological factors and the epidemiological characteristics of 11 respiratory pathogens in children based on respiratory pathogen surveillance data collected in Lanzhou, China, from January 2023 to May 2025. Spearman correlation analysis and the DLNM were employed. The results indicate that IFV, HAdV, RV, and RSV are the predominant pathogens circulating among children in this region. The detection rates of each pathogen exhibited significant age-dependent and pathogen-specific spatiotemporal distribution patterns. DLNM analysis further indicated pathogen-specific exposure-response relationships between meteorological factors and pathogen detection risk, dominated by short-term and instantaneous effects.

Our previous study preliminarily revealed the impact of meteorological factors and air pollutants on adenovirus epidemics among children in Lanzhou (Wang et al., 2025a). Building upon this foundation, the present study expanded to multiple pathogen lineages, further confirming that meteorological influences are not unique to HAdV but broadly affect various respiratory pathogens, with distinct response directions and intensities observed across different pathogens. Compared to previous correlation-based analytical frameworks, the introduction of the DLNM in this study enabled a more refined characterization of nonlinear exposure-response relationships and their lag structures. This provides higher-resolution evidence for understanding the temporal dynamics between environmental exposure and pathogen activity.

Epidemiological stratification results indicate that respiratory pathogen infections among children in Lanzhou exhibit distinct age-specific distribution patterns, consistent with previously established pathogen-specific trends in other regions. RSV and IFV exhibited the highest detection rates among infants (<1 year old), consistent with the findings of Gentile et al (Gentile et al., 2025). and Blümel et al (Blümel et al., 2015). Potential mechanisms may be related to factors such as the immature development of the infant’s immune system, the decline of maternal antibodies with age, and relatively insufficient numbers and functions of dendritic cells. Concurrently, the <1-year-old group predominantly exhibited single infections (35.1%) and the lowest proportion of mixed infections (2.8%). This likely reflects the relatively limited activity ranges and contact networks of infants and toddlers, where pathogen exposure sources are more concentrated within household transmission chains, thereby reducing the probability of simultaneous exposure to multiple pathogens. MP exhibited the highest detection rate among school-aged children (6–11 years), consistent with previous domestic and international studies (Xu et al., 2024; Zhang et al., 2025; Kutty et al., 2019). This pattern aligns with the transmission advantage conferred by frequent close contact in collective settings, such as schools. The proportion of mixed infections increased significantly with age (from 2.8% in the <1-year-old group to 11.6% in the 3–6-year-old group), suggesting that expanding social contact networks and increased exposure heterogeneity may be key drivers of the rising co-infection risk. Previous studies have indicated that MP-associated infections may increase disease severity by modulating host immune responses and exacerbating airway inflammation (Ye and Wang, 2022), highlighting the need for enhanced surveillance and clinical recognition among school-aged children. RV maintained high detection rates across all age groups, peaking in the 1–3-year-old cohort, consistent with findings from Baiyin, Gansu (Wang et al., 2025b), further underscoring its persistent transmission capacity as a common community pathogen. In contrast, HCoV, HBoV, and HMPV exhibited low detection rates (<5%) across all age groups. This may indicate limited local epidemic intensity or a reduced likelihood of clinical presentation and testing due to relatively mild symptoms. The observed age distribution characteristics suggest that immunization and prevention strategies should adopt age-specific stratification, prioritizing enhanced passive immunity against RSV or influenza vaccination in infants and young children.

Gender stratification analysis revealed no statistically significant differences in detection rates of most respiratory pathogens between male and female children. IFV showed similar positivity rates between the sexes, whereas the RSV distribution was nearly identical. No significant sex differences were observed for pathogens such as MP, HPIV, and HBoV. These findings largely align with most previous studies in pediatric populations but partially contradict our earlier report, which indicated higher MP infection rates among male children (Li et al., 2025). This discrepancy may be attributed to factors such as variations in study periods and epidemic intensity, differences in regional population composition, sample size and statistical power, and adjustments in clinical management and testing strategies. This suggests that the influence of sex on MP susceptibility may be context-dependent and warrants further validation in larger-scale, multicenter, or longer-term cohort studies.

Seasonal analysis showed that the distribution patterns of respiratory pathogens largely aligned with those in the previous literature but also exhibited regional differences. IFV demonstrated a typical winter epidemic pattern, whereas EV exhibited a summer peak (18.9%), consistent with its typical epidemiological characteristics. The high-temperature, high-humidity summer environment provides favorable conditions for EV survival (Zhang et al., 2025), while increased outdoor activities and exposure opportunities among children during summer promote viral transmission. Notably, EV maintained considerable activity in autumn (11.2%), potentially related to local climate characteristics and population behavior patterns, suggesting the need for extended enterovirus surveillance periods. The double-peak distribution pattern of RSV warrants further attention. Traditionally, RSV exhibits winter-spring epidemics in temperate regions, consistent with our findings (Decker et al., 2024). However, the complete absence of RSV detection during summer may be related to test sensitivity, sample collection strategies, or unique local climatic conditions. The concurrent circulation of RSV and IFV during winter complicates clinical diagnosis and underscores the need for enhanced multi-pathogen co-detection during peak respiratory disease seasons. RV and HADV exhibited relatively stable year-round circulation patterns, consistent with their biological characteristics as common respiratory pathogens (Moriyama et al., 2020; Monto, 2002). The relative peak in RV prevalence during autumn (12.4%) may be associated with the start of the school year, temperature changes, and increased indoor activity. The stable year-round circulation of HAdV establishes it as an important baseline pathogen for respiratory infections, warranting continuous attention in clinical diagnosis and treatment.

The Spearman correlation matrix systematically revealed associative patterns between major respiratory pathogens and various meteorological factors, demonstrating complex and highly specific correlations between pathogen prevalence and meteorological conditions. Significant internal correlation structures exist among meteorological factors: temperature is negatively correlated with air pressure, whereas humidity is positively correlated with precipitation. This indicates that changes in individual meteorological indicators are rarely isolated events but rather components of broader atmospheric-system fluctuations. Therefore, interpreting the impact of environmental exposure on respiratory pathogens requires careful consideration of the coupling effects of multiple factors. Among the pathogens, HAdV and RV exhibited positive correlations, suggesting that they may share similar seasonal drivers or exhibit common transmission dynamics during specific periods. This does not rule out the possibility of synergistic transmission or shared host susceptibility between pathogens. Similar phenomena have been reported in other regions (Shi et al., 2025; Wang et al., 2025), indicating that further exploration of ecological interaction mechanisms among pathogens, including immune interference, competitive exclusion, and co-infection, is necessary. Regarding the associations between pathogens and meteorological factors, the results revealed highly variable sensitivities to climatic fluctuations across different pathogens. Significant heterogeneity in the sensitivity to meteorological fluctuations was observed among respiratory pathogens. IFV exhibited a significant negative correlation with low temperatures and low precipitation and a positive correlation with higher atmospheric pressure. This aligns with its epidemiological preference for cold, dry, and stable environments, which is consistent with previous findings in Lanzhou (Du et al., 2022). Lower temperatures may enhance viral stability in aerosols and on surfaces while weakening host mucosal barriers and local immune responses, thereby increasing the risk of infection. MP showed positive correlations with relative humidity and barometric pressure, suggesting that it may spread more readily in mild, humid environments with relatively stable atmospheric pressure. This finding aligns with previous reports of heightened MP activity during seasonal transitions or periods of high humidity (Onozuka et al., 2009; Wright et al., 1969). RSV showed negative correlations with temperature, temperature variation, precipitation, and daylight duration, and a positive correlation with atmospheric pressure, further supporting its classification as a classic winter pathogen. HMPV correlates positively with humidity but negatively with temperature variation, suggesting that its transmission may rely more on humid conditions with minimal meteorological fluctuations. From a virological perspective, enveloped viruses (e.g., IFV and RSV) possess lipid-enveloped structures. Low-temperature, low-humidity environments may enhance their environmental survival and transmission efficiency by reducing water activity and increasing their envelope stability (Lowen et al., 2007). The unique climatic conditions of Lanzhou during winter and spring (low temperatures, low humidity, and high atmospheric pressure) favor prolonged viral survival and efficient transmission, explaining the significant epidemic peaks of IFV and RSV during these seasons. Conversely, high temperatures or humidity may disrupt viral lipid envelopes, leading to inactivation, which is consistent with the reduced activity of these pathogens during warm and humid seasons (Noti et al., 2013). Non-enveloped viruses such as RV and HAdV, which possess more robust protein capsid structures, maintain stability across broader temperature and humidity ranges, exhibiting distinct seasonal response patterns (Stanford Environmental Health and Safety, 2025). Furthermore, the correlation matrix delineates the “meteorological niches” of different pathogens across multidimensional scales, suggesting complementary, alternating, or overlapping seasonal prevalence patterns. This provides crucial foundational information for understanding the complex seasonal formation mechanisms and developing integrated multi-pathogen early warning systems.

DLNM results further revealed a significant and pathogen-specific exposure-response relationship between primary meteorological factors and pathogen detection risk, with peak effects almost entirely concentrated at lag 0 weeks. This suggests that meteorological exposure primarily influences pathogen activity through short-term effects that occur within the same week. This phenomenon may result from multiple factors: most respiratory pathogens have short incubation periods, making them more likely to manifest as same-week effects in weekly aggregated data; weekly temporal resolution limits the identification of daily level lag distributions, potentially folding short-term delayed effects into lag 0; detection and reporting processes are typically completed within the same week symptoms appear, potentially shifting observed effects temporally forward; beyond major pathogens, detection rates for some pathogens are low, with statistical power insufficient to detect weaker delayed effects. IFV exhibits a significantly elevated risk under low temperature and high barometric pressure conditions, with particularly pronounced RR values during extreme exposure events. This aligns with the environmental characteristics reported in Guangzhou, China, where cold, dry, and high-pressure conditions promote influenza epidemics (Guo et al., 2019). Low temperatures enhance viral stability in aerosols, whereas high barometric pressure typically correlates with cold, dry, and stable atmospheric conditions, potentially prolonging viral persistence in the environment. The risk of HAdV similarly increases under low temperatures and high-pressure conditions, potentially linked to its environmental tolerance and the meteorological sensitivity of host immune barriers. Despite being a relatively resilient non-enveloped virus, the risk of HAdV detection exhibits pronounced meteorological dependence, suggesting that environmental conditions are not minor factors in its transmission dynamics. MP exhibits bidirectional responsiveness to meteorological conditions. High pressure and moderate temperature ranges were correlated with an elevated MP risk, whereas high wind speeds consistently demonstrated a protective effect. This protective influence likely stems from pathogen dilution via airflow and reduced droplet concentration in enclosed spaces, providing real-world evidence supporting public health strategies that emphasize enhanced ventilation to mitigate respiratory transmission risks.

RSV and HMPV exhibit greater sensitivity to humidity and temperature fluctuations, with RSV posing a higher risk under conditions of low temperatures, reduced sunlight, and lower precipitation. In some cases, RSV exhibited a secondary peak delayed by 1–2 weeks, but its effect size was significantly smaller than the immediate effect, indicating that although a certain delayed effect exists, its prevalence is primarily driven by immediate meteorological conditions. In contrast, HPIV and RV showed protective associations with low temperatures and higher wind speeds. This phenomenon stands in stark contrast to pathogens such as IFV and RSV, suggesting fundamental differences in the “ecological niches” of different pathogens within meteorological factors. RV, a highly tolerant non-enveloped virus, is more likely to be driven by human behavioral patterns (e.g., indoor activities and school-aged gatherings) than environmental survivability. Consequently, the detection rate decreases under colder or windier conditions with reduced outdoor activity. This study comprehensively analyzed the post-epidemic dynamics of respiratory infections in Lanzhou children by integrating epidemiological modeling with demographic and meteorological data. Key strengths include: Long-term monitoring spanning multiple seasons - Simultaneous analysis of 11 major respiratory pathogens within the same pediatric cohort - Application of GAM-DLNM to detect complex, nonlinear, and lagged associations between climate variables and infection rates, providing deeper insights than traditional correlation methods - Age- and setting-specific analyses enabling refined understanding of population susceptibility and seasonal burden Overall, this study revealed highly heterogeneous responses of respiratory pathogens to meteorological conditions: IFV, HAdV, and MP tend to pose a higher risk in environments with low temperatures or high atmospheric pressure; HPIV and RV show protective associations under conditions of low temperature, low humidity, or high wind speed; this suggests that improving ventilation may have the potential to reduce the risk of infection. To gain a more comprehensive understanding of the generalizability and regional specificity of our findings, we conducted a systematic comparison of the epidemiological characteristics of respiratory pathogens in children in Lanzhou with those in studies from different climatic regions, both domestically and internationally. These comparisons indicate that climatic differences significantly influence the seasonal epidemiological patterns of respiratory pathogens; however, the responses of different pathogens to meteorological conditions exhibit a high degree of consistency alongside some regional specificity, consistent with previous research findings (Tang and Loh, 2014; Zheng et al., 2021; Moazeni et al., 2023; Limaheluw et al., 2024).

Experimental studies by Lowen et al (Lowen et al., 2007)in temperate regions demonstrated that IFV transmission efficiency markedly increases under low-temperature and low-humidity conditions, with epidemic peaks predominantly occurring in the winter. The winter-spring IFV peak observed in Lanzhou and its positive correlation with low temperatures and high atmospheric pressure align closely with this classic finding, further validating the cross-regional robustness of IFV’s preference for cold, dry environments (Pica and Bouvier, 2014). However, studies in tropical and subtropical regions indicate that IFV epidemics may exhibit a bimodal pattern or year-round sporadic characteristics (Caini et al., 2015), suggesting that both the absolute values of temperature and humidity and their seasonal fluctuation patterns may jointly shape regional epidemiological patterns of IFV. RSV exhibits pronounced seasonality worldwide (Bashir et al., 2013). Li et al (Li et al., 2024). found in northern China that RSV detection peaks primarily occur from November to February, closely associated with low temperatures and humidity, this is consistent with the findings of the present study and current epidemiological data (Reiche et al., 2014; Darniot et al., 2018). In tropical regions, a global systematic review by Bloom-Feshbach et al (Bloom-Feshbach et al., 2013). indicated that RSV peaks typically coincide with the rainy season, suggesting that RSV seasonal patterns may be driven by different dominant factors across climatic zones, predominantly temperature in temperate regions, versus seasonal variations in precipitation and humidity in tropical areas. This study observed negative correlations between RSV and temperature/sunlight exposure, coupled with a positive correlation with atmospheric pressure, further supporting its stable and endemic pattern as a typical winter pathogen in temperate continental climates. The seasonal characteristics of MP are complex in nature. Xu et al (Xu et al., 2011). found in southern China that MP peaks predominantly occur during summer and autumn, associated with high-temperature and high-humidity environments, whereas studies in the United States report MP activity being more pronounced during autumn, winter, or seasonal transition periods (Hamid et al., 2023)This study found that MP was positively correlated with relative humidity and atmospheric pressure, suggesting a higher transmission efficiency under relatively stable meteorological conditions with moderate humidity. This finding partially supports the hypothesis that MP epidemics are influenced by meteorological fluctuations during seasonal transitions. However, this also indicates that MP epidemics may be regulated by factors beyond meteorology (e.g., population immunity levels and antibiotic usage patterns), leading to significant regional heterogeneity in their epidemiological characteristics. RV is widely recognized as a year-round pathogen that exhibits seasonal preferences. Monitoring by Tang et al (Tang et al., 2025). in Yongzhou, China, showed slightly higher RV detection rates in spring and autumn, which is consistent with our results. This study observed protective associations for RV under low temperatures or high wind speeds, further supporting the notion that its circulation is primarily driven by indoor gathering behavior rather than viral environmental stability. HAdV exhibits diverse circulation patterns in different regions. Some studies have reported summer peaks (Li et al., 2025), whereas others have observed higher incidences during winter and spring (Wang et al., 2025a). This study found an increased HAdV risk at low temperatures and high atmospheric pressure, consistent with the relatively higher incidence of HAdV in Lanzhou during winter and spring. As a non-enveloped virus, HAdV theoretically possesses strong environmental resilience; however, its detection risk is significantly meteorologically dependent. This suggests that seasonal fluctuations in host immune status, indoor gathering behavior, and regional differences in the prevalence of specific serotypes may collectively influence their spatiotemporal distribution. The global epidemiological patterns of HMPV and HPIV are relatively similar to each other. Previous studies have indicated that HMPV primarily circulates during winter and spring, exhibiting a high seasonal overlap with RSV (Xu et al., 2021). This contrasts with the findings of this study, where HMPV showed a positive correlation with humidity and a negative correlation with temperature. However, HPIV exhibits more dispersed seasonality, with different serotypes potentially peaking at different times (Han et al., 2022). In this study, HPIV showed protective associations with low humidity, low temperature, and high wind speed, suggesting that its circulation may not depend on cold, dry environments but is more likely influenced by specific population immune backgrounds and social contact patterns. In summary, our findings strongly align with the international literature on the meteorological sensitivity of major pathogens (e.g., IFV and RSV), validating the cross-regional robustness of the classic epidemiological pattern in which cold, dry conditions promote enveloped virus transmission (Shang et al., 2026). However, pathogens such as MP, RV, and HAdV exhibit more pronounced regional specificity in their epidemiological patterns than do other pathogens. This suggests that factors beyond meteorological conditions, including population immunity levels, social contact networks, healthcare accessibility, and pathogen genotype distribution, may play varying roles across different regions.

This study deepens our understanding of the respiratory infection patterns among children in Lanzhou during the post-pandemic period. However, this study had several limitations. First, its observational design cannot rule out potential unmeasured confounders such as indoor heating, air purification device usage, differences in children’s indoor versus outdoor activity patterns, and seasonal variations in healthcare-seeking behavior. Therefore, while providing strong quantitative evidence supporting an immediate association between meteorological factors and short-term pathogen dynamics, causality cannot be directly inferred and should be regarded as hypothesis-generating research. Second, the weekly resolution of the data limited our ability to capture the daily level lag effects. The concentration of peaks at lag 0 weeks may partially reflect the cumulative effects of pathogens with incubation periods shorter than 7 days within the weekly aggregation. Future studies should validate these findings using daily-level data and explore longer lag periods (e.g., 0–6 weeks) to comprehensively assess the cumulative effects. Furthermore, the exposure-lag-response surface reveals that even when the primary effects of all pathogens cluster at lag 0, meteorological factors still exhibit discernible variations within short-term lag windows. This finding suggests that incorporating pathogen-specific lag structures and meteorological niche characteristics into future infectious disease early warning models could enhance prediction accuracy. Finally, as this study is based on single-center data, the generalizability of these findings requires further validation across multicenter and multi-regional populations. Despite these limitations, this study provides several practical insights for epidemiological understanding and public health policy in Northwest China, particularly offering valuable references for age-stratified prevention strategies, meteorological early warning system development, and ventilation intervention promotion. In summary, these findings provide crucial theoretical and empirical support for incorporating climate variables into respiratory disease prediction models in the Lanzhou area. This study underscores the urgent need to develop geographically adaptive and pathogen-specific public health control strategies, given the significant variations in regional climate characteristics and epidemiological patterns, to effectively reduce the incidence risk of childhood respiratory infectious diseases. This study offers scientific guidance for refining regional disease prevention and control systems.

## Data Availability

The original contributions presented in the study are included in the article/**Supplementary Material**. Further inquiries can be directed to the corresponding author.
